# Supplementary material for: Patterns of Intron Gain and Loss in Fungi
Source: PLoS Biol. 2004 Nov 30;2(12):e422. doi: 10.1371/journal.pbio.0020422 (PMC532390; doi:10.1371/journal.pbio.0020422)
Supplement: Table S1 — Also available at http://genes.mit.edu/NielsenEtAl/. (4.3 MB ZIP). [file pbio.0020422.st001.zip › NielsenEtAl/html/31.html]

AN2985.1.NCU00711.1.MG10469.1.FG03726.1


```
 CLUSTAL W (1.82) Multiple Sequence Alignments - Introns Inserted


Sequence 1: NCU00711.1	603 aa
Sequence 2: MG10469.1	634 aa
Sequence 3: AN2985.1	616 aa
Sequence 4: FG03726.1	543 aa
Alignment Length: 652 aa
Number Identitical Residues: 124 aa
Alignment Score (without introns) 9122


MG10469.1 	MTAPEDAPAQIDAQIRVRSPAVREASSSETGSVSDTVKAMDPCHAPDASIKRTTSSASQH
NCU00711.1	-MASAARGSRGTPAVDSSTLTQTSAAASDNGSSSDNTVQNN-INNPQAKEEQEKEQELER
FG03726.1 	--------------------------MSEPLQSQ---------SQPQSPSHQQDGHNEKD
AN2985.1  	MAAHENPPEEGKPPLDAADMGITTEDLTAPKKAHADESLDD--AAPRSSSSEITSSPQKD
          	  :         .              :   .        .    * :   .      : 

MG10469.1 	PIPAAEPPRAVVGEAVGADATDEEAAVTPPKDHEKSTPPEAGRTKLETTVIVVS~LATAL
NCU00711.1	ANEDADPT---------APPTDPPTTVTGP-----SAAPEEGRTSLETTAIILA~LASAL
FG03726.1 	AVGSRLEP------------VETGASYVPP-----------HMTTLQRRLVVLS~LCLTL
AN2985.1  	DAAAAPDT-----------ETGNPLDRVPS------QAQKLGKKKIAAVMGALC0-----
          	       .            .      . .       . .   ..:      :.      

MG10469.1 	FLAALDISIVTVAVPAIASDFRST-VGYTWIGSAYLLANAAMAPIWGKISDIWGRKQILL
NCU00711.1	FLAALDVTIVTVAIPTISEEFGST-TGYTWIGSAYLLANAATAPMWGKISDIWGRKPILL
FG03726.1 	FLCALDTTILATALPTIGNELQTSAREYAWIGSSYTLSTTAVTPVWAKISDIVGRKPSMM
AN2985.1  	-------TIISTALPTMAVDFNASESGYSWMASSFSLTNAAFVPLWGKISDIWGRKPILL
          	       :*::.*:*::. :: ::   *:*:.*:: *:.:* .*:*.***** ***  ::

MG10469.1 	LAACVFWVGSLLCALSVSMAMLIASRAVQGMGGGGIIIMVNVCISDLFSIR~KRG~VYFG
NCU00711.1	MTVGVFWIGSLICALSKNMGMLLAARAIQGVGGGGIIILVNICISDLFSMR~KRG1IYFG
FG03726.1 	SATGLFMAGSLICALANSSGMLIGGRVVQGLGGGGCMVLITILIGDLFPLK~DRA~KYYG
AN2985.1  	LANLAFLIGSLICALAINLPMVLAGRAIQGAGAGGIITLANISVSDLFSVR2ERP~MYYA
          	 :   *  ***:***: .  *::..*.:** *.** : : .: :.***.:: .*   *:.

MG10469.1 	VMGLVWAVAGAVGPVVGGLFTTKVSWRWCF~YINL~PIAGVGIIILFFVLKLHNPRTPMR
NCU00711.1	VMGMVWALASAIGPILGGTFTSKVTWRWCF~YINL~PISGVGMAILAFVLKLHNPRTPIR
FG03726.1 	ITGLVFGIAGAIGPVLGGVFSQAVTWRWCF1YINL~PFEGVALVALFFLLKVNIEKEPLI
AN2985.1  	LFGSTWAIAGALGPLVGGAFTTSVTWRWCF~YLNL1PIGGFSFLVLFFFLKVDSPKTPLI
          	: * .:.:*.*:**::** *:  *:***** *:** *: *..:  * *.**:.  : *: 

MG10469.1 	QGLAAVDWLGSLTIIAGTVMVLMGLTLG-GVTAPWASPTVLCLLIIGAAMVAVFVLVELR
NCU00711.1	QGLMAVDWLGSVTIVGGTLMVLLGLEFG-GVSYPWKSATVICLIVFGIVVIGIFGLIEWK
FG03726.1 	DGIRSLDWVGFAFIIGGTICFLYGLEAGSGGLAPWNSAFVICLIVFGVVILALFMVWEAK
AN2985.1  	AGLKTIDWSGTLLIVGATLMFLFGLQFG-GVDYPWGSATVLCLIIFGLLTYALAMLNEWK
          	 *: ::** *   *:..*: .* **  * *   ** *. *:**:::*    .:  : * :

MG10469.1 	VAPYPVVPMHLFKVRSNVASLSVAFAHGFTTMSGFYYLPLYFQ~AVLGADALMSGVYILP
NCU00711.1	VAKYPVIPMRLFKRRASVASL---------------------L0AVLGATPLLSGVYVLA
FG03726.1 	VAKNPIIPIRIFQSTTNMASFAVANLHSFIFISYDYFLPLYFQ~VILGFSPIISGLTLFP
AN2985.1  	IARYPVIPIRLFSNWHNVLVLLICFIHSLVFMGGAYYLPFYFQ~SVLLVTPILSGVYVLP
          	:*  *::*:::*.   .:  :  .   .    .     .      :*   .::**: ::.

MG10469.1 	YAVVLSLVSVAAGWYIKKTGKYLPPIIFGFVFLTLGMGLLTDLGGPSPSGPNWAKLMIYT
NCU00711.1	FAMSLSIVSAATGVYIKKTGKYLPPIIFGMTVMTLGFGLFIDLE----PRPNWAKIILFQ
FG03726.1 	LILPMSISTGLGGIFVRKTGNYKIVILIGASLMAIGTGLFISLG----QRKEWVKLVIFQ
AN2985.1  	QVLSLSVLSAMTGVIIKKTGRYRELIVAAFVFLTLGYGLLIDLK----PYASWPRLIIYQ
          	  : :*: :   *  ::***.*   *: .  .:::* **: .*        .* ::::: 

MG10469.1 	~IITGIGVGPNFQSPLIALQTTVAQRDMASATATYQFVRQMSTSISVVVGGVIFQNSMDG
NCU00711.1	2IVAGVGVGPNFQAPLIALQTTVPGRDVAAATGTFGFIRQLSTSVSVVIGGVVFQNRMEK
FG03726.1 	~IIAGLGCGVLFQNPMIALQTHVRQRDMAAAMSAYTFLRGLSVSISIVVGTVLIQRTVRG
AN2985.1  	~LIAGIGTGPLFQAPLVALQANIHPSDMAAGTATFSFLRQVSAAISIVIGTVIYQNMLVD
          	 :::*:* *  ** *::***: :   *:*:. .:: *:* :*.::*:*:* *: *. :  

MG10469.1 	QHARLLERLGPDIAALLTGQDAAASVEAVAAIPGEEGLIARDAFWISLRTMYIVYTVAAG
NCU00711.1	QHERLVQELGPKNASLLTGGSAASNVGRLASLPPDQRQIAREAYFNALRTMYIVYVAFAA
FG03726.1 	GNLTHVSN------------------------DAEQGETDKQGYISGLRVMWAFYTALCG
AN2985.1  	KASTIAAAVGPDTAQSLQNSFAANNHDLIRSLADDQKRVVLSAFTFALSRIWVFYTAIAG
          	         ... :    .  :: .     :   ::     ..:  .*  :: .*.. ..

MG10469.1 	VGLVLCFFIGTRKLSTEHEEHKTGLKTLARVRSKVTGE--------KQEAD--------
NCU00711.1	LGLFVSLFVGSRTLSKEHEEHKTGLDNMKKAKEDAAGEGRRRAPVDEEKAGSR------
FG03726.1 	LMVVSAVFILPKPVKKEASS-ETGTETQQSDKTDAIA----------------------
AN2985.1  	VGLIVSVFVRPVVLSKTHTVAKTGLAEQERARKEILEAQAKARASNNNNKEVPEGKEKV
          	: :. ..*: .  :..     :**       : .         . ....    .....
```
